# Bio-Inspired Evolutionary Model of Spiking Neural Networks in Ionic Liquid Space

**DOI:** 10.3389/fnins.2019.01085

**Published:** 2019-11-08

**Authors:** Ensieh Iranmehr, Saeed Bagheri Shouraki, Mohammad Mahdi Faraji, Nasim Bagheri, Bernabe Linares-Barranco

**Affiliations:** ^1^Artificial Creatures Laboratory, Electrical Engineering Department, Sharif University of Technology, Tehran, Iran; ^2^Instituto de Microelectrońica de Sevilla (CSIC and Univ. de Sevilla), Seville, Spain

**Keywords:** spiking neural network, ionic liquid space, genetic algorithm, evolutionary model, synaptic plasticity, intrinsic plasticity, structural plasticity, liquid state machine

## Abstract

One of the biggest struggles while working with artificial neural networks is being able to come up with models which closely match biological observations. Biological neural networks seem to capable of creating and pruning dendritic spines, leading to synapses being changed, which results in higher learning capability. The latter forms the basis of the present study in which a new ionic model for reservoir-like networks, consisting of spiking neurons, is introduced. High plasticity of this model makes learning possible with a fewer number of neurons. In order to study the effect of the applied stimulus in an ionic liquid space through time, a diffusion operator is used which somehow compensates for the separation between spatial and temporal coding in spiking neural networks and therefore, makes the mentioned model suitable for spatiotemporal patterns. Inspired by partial structural changes in the human brain over the years, the proposed model evolves during the learning process. The effect of topological evolution on the proposed model's performance for some classification problems is studied in this paper. Several datasets have been used to evaluate the performance of the proposed model compared to the original LSM. Classification results via separation and accuracy values have shown that the proposed ionic liquid outperforms the original LSM.

## 1. Introduction

Artificial Intelligence, also known as AI, is intelligence demonstrated by machines. Also an area of computer science, AI is one of the most developed scientific fields which has brought so much attention to itself over the past few years. Despite the remarkable development of AI systems over the past few years, designing a system which holds the capabilities of the human brain seems rather hard to achieve. Many AI-based computational systems have been developed so far; yet, none of them can compare to the processing mechanism of the human brain. Designing intelligent systems with the ability to carry out computations similar to the way the human brain does, is studied in the fields of neural networks and fuzzy systems.

Since the most common data obtained from the brain's response to external stimuli are spatiotemporal data, the brain is considered to be a spatiotemporal-data-processing machine. Despite the development of precise brain models (Markram, 2006; Toga et al., 2006; Izhikevich and Edelman, 2008), the available models cannot be used for machine learning and or recognizing spatiotemporal patterns, since they are designed to model the brain's structure as well as its function and, therefore, are unable to mine and learn from the brain data. Creating an integrated computational structure in order to process spatiotemporal data is therefore, one of the main challenges of brain simulation.

Spiking neural networks seem suitable for creating such structures since they are designed to process spiking information which creates spatiotemporal data. In other words, considering the fact that there is information at the exact time a spike appears, spiking neurons send out the information via spikes instead of firing rates (Haykin, 1998). Research shows that temporal coding is used to represent and process the information in brain cortex (Ikegaya et al., 2004; Butts et al., 2007). Besides, spiking neurons are more dynamic since they use time domain (Rolls and Tovee, 1995). Hence, in this paper, spiking neural networks are used to create a computational structure for both comprehending and learning spatiotemporal data.

A large amount of data is processed in the neocortex via stereotypical neural micro circuitry. Therefore, the introduced model is based on the idea of Dynamic Reservoir Networks (DRN) (Schrauwen et al., 2007), i.e., Liquid State Machines (LSM) and Echo State Networks (ESN) (Jaeger, 2001; Maass et al., 2002; Natschläger et al., 2002), which are examples of recursive neural networks with a random structure. The natural dynamic of these networks makes them suitable for online processing of time variant inputs. The mentioned reservoir networks receive the input streams and transform them into non-linear patterns in higher dimensions and show a fading memory of the recent inputs. The reservoir acts as a filter. The state of the reservoir or state vector is then used as inputs for the readout layer. The readout layer of the mentioned networks is used for online processing of the time series. This layer needs to be trained using some simple algorithms such as linear regression methods. The dynamic of the reservoirs directly affects the performance of these networks.

A random generated liquid may not act as a useful filter. Researchers are therefore forced to generate many random liquids until a useful filter is found. On the other hand, there are a lot of studies on improving the liquid in order to find a more useful filter (Lazar et al., 2007; Norton and Ventura, 2010; Rhéaume et al., 2011; Hazan and Manevitz, 2012; Notley and Gruning, 2012; Wojcik, 2012; Hourdakis and Trahanias, 2013; Ju et al., 2013; Sillin et al., 2013; Xue et al., 2013; Roy and Basu, 2016). These studies focus on enhancing the liquid performance by applying different learning rules or neuron models to the liquid. For example Norton and Ventura (2010), proposed a learning rule for training the liquid of LSM in order to construct a suitable liquid, Roy and Basu (2016) proposed an online structural plasticity rule to generate better liquids, Hourdakis and Trahanias (2013) used a measure in an evolutionary framework to generate liquid with appropriate parameters and Wojcik (2012) used Hodgkin and Huxley neurons instead of LIF neurons.

This paper proposes an ionic model of reservoir-like networks in which spiking neurons are located. Connections between spiking neurons are provided by ionic density. One of the exciting aspects of this model is that the link to ion fields invokes an abstraction of biologically plausible processes which may set a foundation for possible future research into neural network dynamics, integrating both spiking and field-based computation in biology. Since all neurons in the ionic liquid can connect to each other, the introduced model can be considered a recursive network. In an ionic space, ionic diffusion is one of the most important factors which affects the connection between neurons. Several algorithms have been developed based on diffusion, including Active Learning Fuzzy Modeling Method (ALM) (Shouraki and Honda, 1997, 1999; Murakami and Honda, 2007). In ALM, features are extracted using an operator known as Ink Drop Spread (IDS) (Murakami and Honda, 2007) which is mainly based on diffusion.

Both synaptic and non-synaptic plasticity make the brain capable of learning. A synapse's ability to change in strength over time is known as synaptic plasticity while intrinsic plasticity involves changes in the electrical properties within a single neuron. The contribution between these two different types of plasticity and how it results in the dynamic and the structure of the cortical network, leads to the development of cortical information processing and coding theory. Hebb's rule (Hebb, 1949) is a learning method based on biological observations which form the basis of “memory” and “learning.” So far, many learning methods (Bienenstock et al., 1982; Oja, 1982; Song et al., 2000; Bi and Poo, 2001; Panchev and Wermter, 2004; Ponulak, 2005; Ponulak and Kasiński, 2010; Mohemmed et al., 2012) have been developed based on Hebb's rule such as the well-known Spike-timing-dependent plasticity (STDP).

Also, Lazar et al. (2007) shows how the computational capability of LSM can be improved by combining STDP and Intrinsic Plasticity (IP). Hence, in the introduced model, intrinsic plasticity has been used to maintain the homeostasis of neuronal performance (Desai et al., 1999; Daoudal and Debanne, 2003). Homeostasis is in fact a biological term describing the stability of the environment in which cells exist and is defined as the will to create an almost-stable balance between dependent elements. A lot of learning rules for training networks of spiking neurons are proposed through various forms of synaptic plasticity (Ponulak and Kasiński, 2010; Kuhlmann et al., 2013; Sporea and Grüning, 2013; Gardner et al., 2015). Most of them have explored weight plasticity which refers to modifying the synaptic strengths. Diehl and Cook (2016) have shown that inclusion of synaptic plasticity within reservoirs can help in learning and inferring relationships between inputs. Also, Panda and Roy (2017) have shown that inclusion of Hebbian and non-Hebbian plasticity at the same time helps in the learning of stable contextual dependencies between temporal sequences. There is another form of plasticity, known as structural plasticity, which is explored in a few works (Roy et al., 2016; Roy and Basu, 2017). This form of plasticity revolves around training a network through formation and elimination of synapses. Recently, structural plasticity has been used to train feed forward neural networks (George et al., 2015b), recurrent neural networks (George et al., 2015a), and reservoir networks (Roy and Basu, 2016). The proposed model therefore uses synaptic plasticity caused by dendritic spines (known as dendritic plasticity) to improve learning (Roberts et al., 2010; Tschida and Mooney, 2012). Dendritic spines increase the learning ability in humans by creating and removing synapses. The disability to learn in children suffering from Down Syndrome and/or Fragile X Syndrome is in fact due to the weakness of dendritic spines (Wang et al., 2012). The proposed model therefore tries to create new synapses and removes the pre-existing ones by considering the movement of dendrites, which somehow leads to improving the ability to learn with fewer neurons.

This paper, just like other works, focuses on improving the liquid through structural plasticity (Roy and Basu, 2016) and intrinsic plasticity (Lazar et al., 2007) but with a major difference. Training in the proposed model is performed through ionic liquid. Ionic liquid makes structural plasticity as well as weight plasticity possible, using diffusion, which induces online learning due to simple computations. Both of these types of plasticity are motivated from biological observations.

The proposed model seems to be a better way to model reservoirs because of the following two advantages: (1) The proposed connectivity, which is provided by diffusion in the ionic space, is one of the few dynamic connectivity models that does not involve complex computations. (2) Since the connectivity information is transmitted locally through the ion field, none of the neurons require any knowledge of the global state to update their connections.

The human brain has evolved over more than five million years. Therefore, proposing precise models of the brain requires modeling evolution basics in nature. Based on biological findings, the human brain has evolved over time and therefore, we have tried improving the efficiency of the introduced model using evolutionary algorithms (Simon, 2013). Evolutionary algorithms such as the Genetic Algorithm (GA) and Particle Swarm Optimization (PSO) have several applications in machine learning (Faraji et al., 2017; Iranmehr et al., 2017) and neural networks (Gao et al., 2019). In this paper, the topology of the introduced model is optimized using a genetic algorithm as well as some experiments. For each problem, the topology of the introduced model can be optimized so that it would have the best performance. The term “optimizing the topology” in this paper can refer to optimizing the total number of network's neurons, number of dendrites per neuron and the location of dendrites and axons' terminals. In other words, in this paper, in order to solve a particular problem, we try to optimize the topology of the introduced model so that the efficiency increases. Since the proposed model is suitable for classifying spatiotemporal data, its performance when classifying some time-varying datasets has been analyzed.

Concisely, this paper proposes a new model of reservoir networks where the neural dynamics are modeled by the ionic diffusion formulae and also focuses on topologic optimization of the introduced model using evolutionary algorithms in order to improve its performance. The proposed model which is suitable for processing spatiotemporal or time varying data has been used in classifying different datasets such as N-MNIST (Orchard et al., 2015), TIMIT (Garofolo et al., 1993), and FSDD (Jackson, 2016). In section 2, we discuss the introduced model and provide information about the structure of the proposed model, the ionic space in which the neurons are located, and ionic diffusion which leads to neuronal connections being made. Section 3 studies the performance of the proposed model using different metrics such as separation, approximation and generalization. Section 4 focuses on optimizing the topology of the introduced model. Section 5 includes the results obtained from evaluating the classification performance of the proposed neural network on the N-MNIST dataset. Moreover, the results obtained from comparing our proposed model with some other works are included in section 5. Finally, we conclude the paper in section 6.

## 2. The Proposed Spiking Neural Network in Ionic Liquid Space

Here, a computational model based on spiking neurons is introduced, considering biological observations as well as reservoir networks. Since the electrochemical transition of neurons occurs via ions, it is assumed that the neurons are placed in an ionic liquid space which we refer to as ILS. The density of ions in the ionic liquid space strengthens some of the connections between neurons and allows neurons to connect to each other via the ionic liquid. In conventional neural networks, each synapse is modeled by a coefficient. However, in the introduced model, the synapses are modeled using ionic diffusion in ILS. Since the main factor in diffusion is time delay, it can be said that in the introduced model, synapses are somehow modeled dynamically. In this section, after explaining the structure of the introduced computational model, we describe the network, considering both intrinsic and synaptic plasticity.

### 2.1. Structure of the Proposed Computational Model

**Figure 1:**
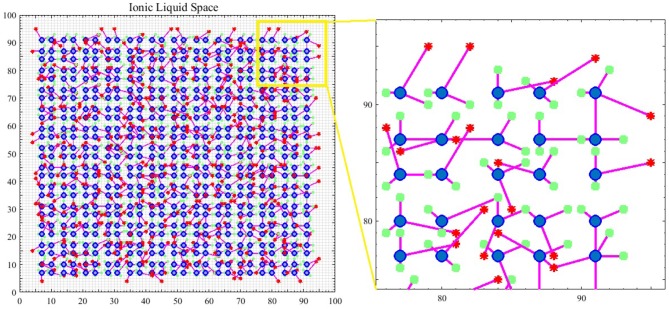
An example of a spiking neural network in ionic liquid space. A total number of 25 × 25 = 625 spiking neurons are placed in a 100 × 100 ILS. In this example, each neuron has two dendrites and one axon. In this figure, neurons are depicted by blue circles while the location of dendrites and the terminals of axons are depicted by green squares and red stars, respectively. A small area of the ILS is zoomed in on and depicted in this figure. It can also be seen that in the proposed model, the neurons are not directly connected and instead, are connected via ILS.

What leads neurons to respond to stimuli and transmit spikes is the imbalanced distribution of ions in the intracellular and extracellular space of nerve cell membranes. Therefore, in the introduced model, it is assumed that spiking neurons are placed in an ionic liquid space. The mentioned liquid space can be either two-dimensional or three-dimensional based on the defined problem. To simplify, the ionic liquid space is considered to be two-dimensional in the present paper. The ion density gradient, which is due to the presence of stimulus in this space, leads to diffusion since ions move from a point with higher concentration to a point with lower concentration. The difference in ion density leads to voltage difference. Furthermore, ions diffuse through cell membrane at a molecular level. Therefore, in order to model the diffusion process, we first need to quantize the ILS to a particular number of bins and then, assign a voltage level to the ion density of each bin. Diffusion in the ILS causes the voltage level of each bin to change over time. Figure 1 shows an example of the computational model introduced in this paper. The 2*D* ionic liquid space is quantized to 100 × 100 bins. In this ILS, there are 25 × 25 = 625 neurons which are depicted in blue. Each neuron consists of two dendrites and one axon which are depicted in magenta. The terminals of axons and dendrites are depicted with red stars and green squares, respectively. The terminals of axons and dendrites are each placed in a bin so that the dendrites can receive the bin voltage, transmit it to the soma and also, to enter the output spike into a bin from the ionic liquid space via axons at the time a neuron is fired. External stimulus can be applied to each of the bins of ILS which leads to an increase in voltage in that bin. The difference in voltage between the mentioned bin and its neighbor leads to diffusion. In order to model the diffusion process in this ionic liquid space, it is assumed that each bin located in (*x, y*) in the time step *t* holds a voltage equal to $V_{xy}^{t}$. Considering all eight neighborhoods of the mentioned bin, we can model the diffusion process by Equation (1) in which α is a coefficient representing the transmitted current between bins and β is another coefficient which keeps the ionic liquid space from saturating.

(1) $$\begin{array}{r} V_{xy}^{t+1}=\left(1-\beta \right)\times V_{xy}^{t}-\alpha \overset{1}{\underset{m=-1}{\sum }}\overset{1}{\underset{n=-1}{\sum }}\left(V_{xy}^{t}-V_{\left(x+m\right)\left(y+n\right)}^{t}\right) \end{array}$$

Due to the difference in ionic density in the ionic liquid space which leads to voltage difference among different bins, ion trajectories are created. These trajectories depend on the applied stimuli and the diffusion process. In other words, each applied stimulus creates a different ion trajectory in the ionic liquid space. The created trajectories cause the neurons to activate and therefore, lead to the creation of connections between neurons. Ion trajectories can gradually crystalize or even fade. Once an ion trajectory crystalizes, it strengthens. The fading of an ion trajectory on the other hand, basically means that it gets weaker. Therefore, the connection between neurons in the introduced model is different from that of a conventional neural network. In the introduced model, the connections are made via the ionic space and the created trajectories. It could be understood that every single neuron in the ionic liquid space is capable of making connections with other neurons depending on the created trajectory.

At first, there are no proper connections between neurons in the ionic liquid space just like in regular neural networks in which the weights are initialized randomly. As the diffusion process begins, proper connections begin to develop between neurons. The connection topology of neurons in this ionic liquid space is similar to natural structures as well as brain structures (Zuo et al., 2017). In this topology, neurons are more likely to connect to neighboring neurons. So far, the general structure of the proposed computational model based on spiking neurons has been explained. The following discusses the model of spiking neurons in the ionic liquid space.

The model provided for a spiking neuron represents the creation of an action potential and the effect of neuron inputs on its membrane potential. Several models have been introduced for spiking neurons. The basic idea behind defining spiking neurons is electrical conductance. One of the most well-known electrical models for spiking neurons was introduced by Hodgkin and Huxley (1952). In this model, the electrochemical data transmitted between neurons has been modeled using an electrical circuit consisting of resistors and capacitors (Hodgkin and Huxley, 1952). This model has a high computational cost. Another model which balances computational cost with how close the model is to biological observations, is the Izhikevich model (Izhikevich, 2003). The Leaky Integrate and Fire (LIF) model (Stein, 1965; Abbott, 1999; Gerstner and Kistler, 2002) is derived from Hodgkin-Huxley's model and has, in comparison, a lower computational cost. In the LIF model, each spike represents a uniform event which is defined at the time a spike is created. Due to its simplicity and low computational cost, the LIF model has been used in the structure of the proposed model.

### 2.2. Description of the Proposed Computational Model

Learning capacity of a neural network depends on the plasticity of synapses. In other words, synaptic plasticity is much more important than the number of synapses and or the connections. Due to the fact that the neurons are not directly connected in the proposed model, the introduced network has high plasticity. In fact, the high plasticity of the proposed model is due to the possibility of recreation and or pruning of synapses.

Another effective factor in a network's learning is the intrinsic plasticity that keeps the performance of neurons in a relatively stable equilibrium. While describing the proposed model the intrinsic plasticity should be considered in a way that all neurons can activate. To do so, the firing threshold of neurons in the ionic liquid space should be changed in each time step.

Assuming that the number of spiking neurons in a network is N, the activity of the *ith* neuron in the time step *t* is represented by *x*_*i*_(*t*). If the *ith* neuron is fired, *x*_*i*_(*t*) = 1, while *x*_*i*_(*t*) = 0 for when it is not fired. Each neuron in the ionic liquid space has a specific number of dendrites through which the inputs are entered and an axon through which the output is transferred. If the weighting factor of the *jth* dendrite of the *ith* neuron is represented by *w*_*ij*_ and all connections are considered to be excitatory, the pre-activation value of the *ith* neuron in *t* + 1 time step which is represented by *p*_*i*_(*t* + 1), can be obtained from Equation (2). It should be noted that in the proposed network, excitatory connections mean having positive weights (*w*_*ij*_ ≥ 0). In this equation, *V*^*t*^(*i, j*) represents the voltage of a bin in ILS where the *jth* dendrite of the *ith* neuron is located in the time step *t*. ν_*i*_(*t*) represents the threshold applied to the *ith* neuron in the time step *t* in order to maintain relatively stable equilibrium of neuronal activity. After finding the pre-activation value of all neurons in a specific time step, the first *k* neurons with the highest pre-activation values are obtained using the k-winner-take-all function (Maass, 2000). The activity status of these *k* neurons is set to 1 while the activity of the other *N* − *k* neurons is set to 0. The k-winner-take-all function is represented by kWTA and the activity of neurons is given by Equation (3). In this equation, *X* is a state vector representing the activities of all neurons while *P* is a vector representing their pre-activation values. In this paper, ν_*i*_ is adjusted using a simple model in Lazar et al. (2007). In this simple model, ν_*i*_ is set in a way that the threshold applied to an active neuron increases while it decreases for a deactivated neuron. As a result, the active neuron will need more membrane potential to re-activate while the deactivated neuron will need less. Therefore, more neurons can be active in the proposed model. This leads to higher dynamicity of the introduced model. Equation (4) represents the simple method used to set the value of ν_*i*_. In the given equation, η is a coefficient representing intrinsic plasticity rate.

(2) $$\begin{array}{r} p_{i}\left(t+1\right)=\underset{j}{\sum }\left(w_{ij}\times V^{t}\left(i,j\right)\right)-\nu_{i}\left(t\right) \end{array}$$

(3) $$\begin{array}{r} X\left(t+1\right)=kWTA\left(P\left(t+1\right)\right) \end{array}$$

(4) $$\begin{array}{r} \nu_{i}\left(t+1\right)=\nu_{i}\left(t\right)+\eta \left(x_{i}\left(t\right)-\frac{k}{N}\right) \end{array}$$

### 2.3. An Example to Show Diffusion in ILS and the Activity of Its Neurons

**Figure 2:**
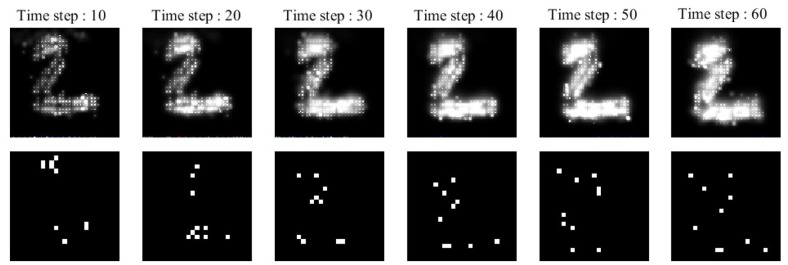
A simple example to show how the proposed model learns a sample of the N-MNIST dataset. Each sample with a duration of about 300 *ms*, is injected into the ionic liquid space through time steps. **Upper row:** represents the diffusion effects on the voltage level of ionic liquid space at six different time steps. **Lower row:** represents the activity of neurons inside ionic liquid space considering *k* = 10. Each sample activates different neurons at different time steps which leads to better separation between samples of different classes.

A simple example, depicted in Figure 2, is employed to explain the diffusion process as well as neuronal activities in the ionic liquid space, based on the proposed model which we described by considering the intrinsic plasticity. In this example, higher voltage levels are depicted in light colors while lower voltage levels are shown in dark colors. The topology of the proposed network's model is similar to the one presented in Figure 1. The term “topology” refers to the local structure of neurons in the ionic liquid space as well as the number of bins on this plane, the number of neurons and the number of dendrites per neuron. In this example, the N-MNIST dataset is used. Every sample of this dataset, which holds a duration of about 300 ms, has been injected into the ionic liquid space over time. In the first row of Figure 2, the voltage level of the ionic liquid space at six different time steps is depicted. The second row represents the activity of neurons in the ionic liquid space. Active neurons are depicted in white while the deactivated neurons are shown in black. Using the description provided for the network in section 2.2, *k* is set to 10 arbitrarily. As mentioned before, *k* represents the number of active neurons of the network in each time step. Having a close look at Figure 2, it can be understood that in each time step, 10 of the neurons in the ILS are active while the rest are deactivated.

The N-MNIST dataset (Orchard et al., 2015) is in fact the neuromorphic version of the MNIST dataset (LeCun et al., 1998). Each sample from this dataset represents a number between 0 and 9. The N-MNIST dataset uses a biological idea known as saccades. Each sample from the MNIST dataset is displayed on a monitor and then, recorded by a motor-driven DVS camera, moving in three different directions in a triangular form. The duration of each N-MNIST sample is about 300 ms. Since the size of each N-MNIST picture is 34 × 34 pixels, the intensity of each pixel is applied to a bin in an ionic liquid space with a total number of 100 × 100 bins. The mentioned mapping is one to one. In this paper, in order to map each pixel to a bin in an ILS, the ILS is divided to regions overlaying one another. The number of these regions is equal to the number of pixels in the picture. Each pixel is randomly mapped to a bin in the region associated with it in ionic liquid space. Once the image is mapped to ionic liquid space in each time step, the voltage level of bins in ionic liquid space changes. Diffusion too leads to changes in the voltage level. The changes in voltage lead to changes in the activity of neurons. For each sample, different neurons in ionic liquid space are activated through time steps which results in more separations between samples of different classes.

## 3. Evaluating the Performance of the Proposed Model

In this section, the performance of the proposed model is studied using popular metrics including separation, approximation, and generalization. Separation is a metric used to determine the effectiveness of the liquid which addresses the amount of separation between state vectors that are caused by different input streams. Approximation is the capability to distinguish and transform different state vectors into the given target outputs. This section also contains an example to show the performance of the proposed model in classification problems using a sound dataset.

### 3.1. Separation, Approximation, and Generalization

One of the methods used for measuring separation is pairwise separation considered in Maass et al. (2002) in which the separation between two different spike trains is computed as Equation (5). In this equation, the ILS internal states at the time *t* are represented by *X*_*u*_(*t*) and *X*_*v*_(*t*) in which *u*(.) and *v*(.) are two spike trains and ||.||_*k*_ denotes the *L*_*k*_ norm.

(5) $$\begin{array}{r} PairwiseSeparation=\underset{t}{\sum }\left(||X_{u}\left(t\right)-X_{v}\left(t\right)|{|}_{2}\right) \end{array}$$

**Figure 3:**
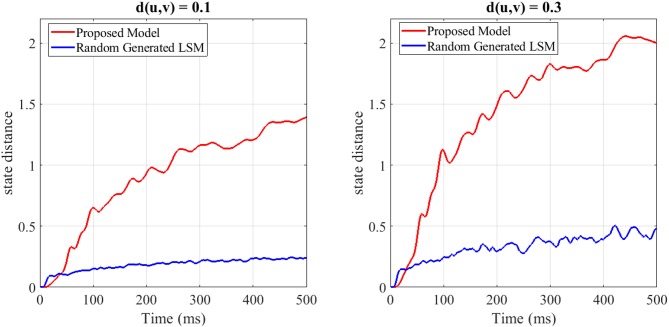
The average state distance as a function of time for two different input distances *d*(*u, v*) = 0.1, 0.3 and for both the random generated liquid and the proposed liquid. It is clearly seen that the proposed liquid outperforms the random generated liquid.

To show the separation capability of the proposed model in comparison with a random generated liquid, an experiment was designed. In this experiment, 200 different spike train pairs *u*(.) and *v*(.) with the length of 500*ms* are generated and given as input to both the random generated liquid and the proposed liquid. Both of the liquids consist of *N* = 25 neurons for a fair comparison. The internal state distance separation averaged over 200 trials for two different input distances *d*(*u, v*) = 0.1, 0.3 is plotted in Figure 3. The proposed liquid and random generated LSM specification are brought in **Table 4**. This figure clearly shows the better separation capability of the proposed model in comparison with the random generated liquid. It can also be seen that more input distance causes more state distance in both of these liquids. To define distance *d*(*u, v*) between the spike train pairs *u*(.) and *v*(.), each spike in the spike trains was replaced by a Gaussian kernel and *d*(*u, v*) was then determined by the distance between the resulting continuous functions.

Another separation measure devised by Goodman and Ventura (2006) is based on the mean distance between the state vectors obtained from each of the classes in a problem. Norton and Ventura (2010) revised Goodman's definition of separation by adding the variance between state vectors of the same class. In Norton's definition of separation, the separation is divided into two parts: inter-class distance *c*_*d*_ and intra-class variance *c*_*v*_. Assuming that *n* is the total number of classes, a set of state vectors *X* is divided into *n* subsets, *X*_*l*_, one for each class. *x* represents an individual state vector. Inter-class distance *c*_*d*_ is defined by Equation (6) in which μ(*X*_*l*_) is the center of mass for each class. It is calculated by Equation (7) where |.| denotes the set cardinality. Also, the mean variance of state vectors of every class *c*_*v*_ is defined by Equation (8) in which ρ(*X*_*l*_) is the average variance of each state vector *x*_*m*_ within the class *l* from the center of mass for that class μ(*X*_*l*_). It is calculated by Equation (9). Then, the separation between *n* input classes can be defined using Equation (10).

(6) $$\begin{array}{r} c_{d}=\overset{n}{\underset{l=1}{\sum }}\overset{n}{\underset{m=1}{\sum }}\frac{||\mu \left(X_{l}\right)-\mu \left(X_{m}\right)|{|}_{2}}{n^{2}} \end{array}$$

(7) $$\begin{array}{r} \mu \left(X_{l}\right)=\frac{\sum_{x_{m}\in X_{l}}x_{m}}{\left|X_{l}\right|} \end{array}$$

(8) $$\begin{array}{r} c_{v}=\frac{1}{n}\overset{n}{\underset{l=1}{\sum }}\rho \left(X_{l}\right) \end{array}$$

(9) $$\begin{array}{r} \rho \left(X_{l}\right)=\frac{\sum_{x_{m}\in X_{l}}||\mu \left(X_{l}\right)-x_{m}|{|}_{2}}{\left|X_{l}\right|} \end{array}$$

(10) $$\begin{array}{r} Sep\left(X\right)=\frac{c_{d}}{c_{v}+1} \end{array}$$

A stronger measure (linear separation) was proposed by Maass et al. (2005) in order to determine whether a readout would be able to produce given target outputs for the input streams. To evaluate the linear separation property of a liquid of *N* neurons for *m* different input streams, the rank of the *N* × *m* matrix *M* has to be computed. The column *m* of matrix *M* is the state vector *X*_*u*_*m*__(*t*) of the input stream *u*_*m*_ at the specific time (*t*). The rank of the matrix (*r*) is a measure to show the computational power of the liquid as well as the number of degrees of freedom that a linear readout has in assigning target outputs to the inputs. Higher *r* corresponds to more computational power or kernel quality. The readout maps are drawn from a class of functions satisfying the approximation property^1^. Besides the separation and the approximation capabilities, the generalization to non-seen or noisy inputs noticeably affects the performance of the model. Maass et al. (2005) quantifies the generalization capability in terms of VC-dimension of the class of hypothesis (*H*) (input-output map). The lower the VC dimension than the size of training set (*S*_*train*_), the greater the generalization capability. Maass et al. (2005) showed the difference of the kernel quality and the VC-dimension (*H*) measures predicting overall computational performance.

To show the generalization capability of the proposed model, the performance of the proposed model for spike pattern classification is computed. In this experiment, we want to classify the spike patterns into two classes using a linear readout. Eighty spike patterns each consisting of 4 Poisson spike trains at 20*Hz* over 200*ms* are considered as input data. The noisy variations of these spike patterns built with Gaussian jitter with 10*ms* standard deviation are used as test samples. The linear readout, trained by linear regression with 500 training samples, is responsible for classifying the test samples. The fraction of test samples (200) which are correctly classified determines the correctness. It should be noted that 20 target classification functions from 2^80^ possible classification functions are selected randomly and their correctness averaged. Classification of these spike patterns using the proposed model results in a mean correctness of 0.71 which is comparable with the correctness achieved by LSM in Maass et al. (2005). It is concluded that the proposed model has both the approximation and the generalization capabilities.

### 3.2. An Example to Show the Performance of the Proposed Model

**Figure 4:**
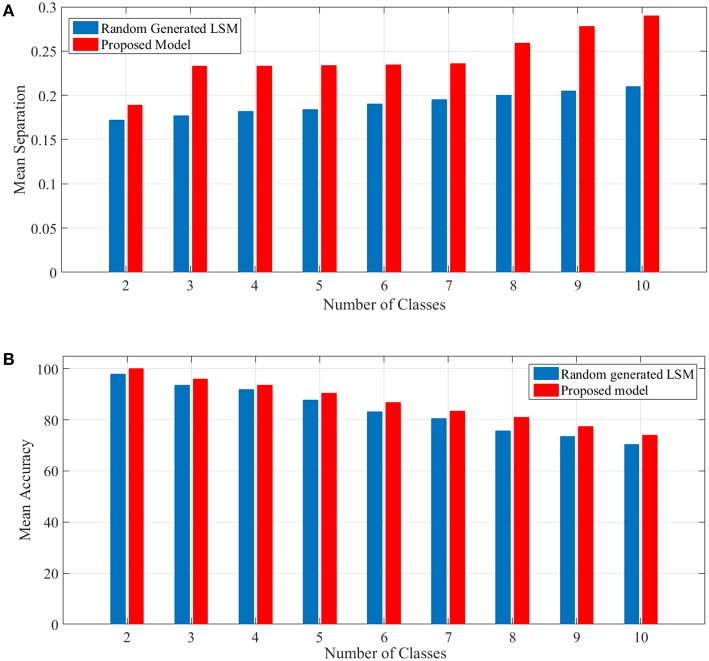
**(A)** Mean separation of the proposed model in comparison with that of the random generated LSM for spoken digit problem using FSDD dataset. Separation considering different number of classes are shown. It is clearly seen that the separation capability of the proposed liquid is better than that of the random generated LSM. **(B)** Mean accuracy of the proposed model in comparison with that of the random generated LSM for spoken digit classification using FSDD dataset.

To show the proposed liquid's separation property for between more than two classes of input streams, we use Norton's definition of separation for identifying free spoken digit dataset (FSDD) (Jackson, 2016) as a second test. This dataset consists of 2000 recordings of digits 0 through 9 (10 classes) obtained from 4 speakers, sampled at 8*kHz*. To transform the digit WAV files into spike trains, first, each digit WAV file is converted into its 49 Mel frequency cepstral coefficients (MFCCs) which are then converted into spike trains. For generating spike trains, we use a method brought in Goodman and Ventura (2006). The spike trains are then injected into the random generated LSM and the proposed liquid for which the specifications are brought in **Table 4** and their separations are obtained. A comparison of mean separation between the proposed liquid and random generated liquid is shown in Figure 4A. Better separation capability of the proposed liquid compared to the random generated liquid can be clearly seen from Figure 4A.

In this experiment, 80% of data which are selected randomly, are used as the training set and the remaining data are used for computing the accuracy. To compute the accuracy, a simple universal approximators consisting of a single layer of perceptrons is considered as the readout layer such as Maass et al. (2002). The readout layer has several modules equal to the number of classes intended. For training the readout layer, the p-delta rule (Auer et al., 2008) is used. The mean accuracy shown in Figure 4B depicts the outperformance of the proposed model compared to the random generated LSM. It is worth mentioning that the results are achieved by considering the problems for 50 liquids either generated randomly for LSM or the proposed model. Since the accuracy was computed using test (unseen) data, the generalization capability of the proposed model was somehow shown by this example.

## 4. Optimizing the Network Topology in Ionic Liquid Space

In this section, the topological parameters of the proposed neural network in ionic liquid space are optimized using an evolutionary algorithm in a way that higher classification accuracy is achieved. To do so, some parameters need to be fixed first. Assuming that the number of neurons in ionic liquid space and also the number of ILS bins are fixed, the optimized number of dendrites per neuron can be obtained by doing some trials each of which is performed for a specific number of dendrites for each neuron in ILS. After optimizing the number of dendrites for each neuron, the location of dendrites and axon terminals are optimized using genetic algorithm (GA).

**Figure 5:**
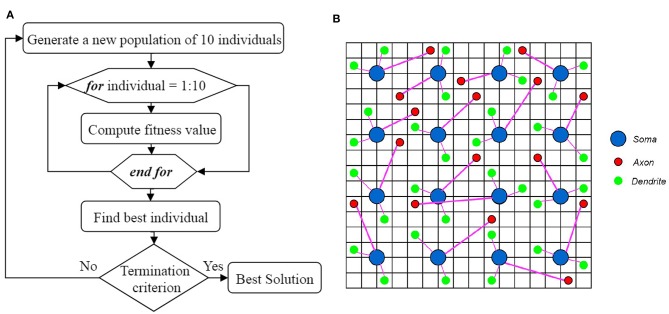
**(A)** The overall process of finding the best solution. **(B)** A topology of a neural network in ILS, represented by an individual of a generation. The terminal locations of axons and dendrites of all the neurons in ILS which are shown by red and green circles respectively, are used to define each individual. Considering that this individual is the best in its generation, the terminal coordinates of axons and dendrites in the next generation of individuals are updated by randomly moving the red and green circles to the neighboring locations.

In genetic algorithm, first, a population of individuals is generated based on some presumptions. This population of individuals (generation) starts to evolve toward a better solution. Figure 5A shows the overall process of finding the best solution using the proposed genetic algorithm. In each generation, the individuals have to be evaluated using a fitness function. The best individual of each generation is obtained based on the fitness values computed using the fitness function. The next generation of individuals is generated using the best individual of each generation. The best individual in all generations is then considered as the best solution. In order to optimize the topology of the neural network in ILS, the individuals need to be represented first and then, a fitness function must be defined to evaluate each individual.

In this paper, some presumptions, including the number of dendrites per neuron as well as the length range of dendrites and axons, are obtained through trial and error. By applying these presumptions, we can reduce the number of parameters to be optimized in the proposed model. By changing these parameters, a population of individuals can be generated. In this paper, changing the location of dendrites and axons in ILS results in the creation of an individual. An individual which is a topology of a neural network in ILS, is depicted in Figure 5B. In this figure, the ILS consists of 16 × 16 bins and 16 neurons. Each neuron consists of 2 dendrites and 1 axon. The neurons are fixed at the specified locations. Each individual is specified by the locations of the terminals of axons and dendrites which are represented by red and green circles, respectively. Considering the fact that each individual has its own unique chromosome, each individual can be represented by its chromosome. In the N-MNIST classification problem in which we have 25 × 25 neurons in ionic liquid, considering that each neuron consists of 2 dendrites and 1 axon, an individual or a chromosome owns a total number of (2 + 1) × 25 × 25 = 1875 genes. In this problem, the first generation consisting of 10 individuals is generated randomly. It should be noted that a simple version of the genetic algorithm shown in Algorithm 1 has been employed for this problem. In this algorithm, in order to generate a new population of individuals, the best individual of the current generation has to be chosen. The best individual in a generation is the one with the highest fitness value. In this paper, the best individual is a topology of the proposed neural network in ILS which leads to higher classification accuracy. Using the best individual, a population of 10 individuals for the next generation is then generated. Since each individual is represented by terminal locations of axons and dendrites of all the neurons in ILS as shown in Figure 5B, the next generation of individuals is generated once the red and green circles move to their neighboring locations. This makes the individuals of the next generation only slightly different from those of the current generation. By considering this simple model, we no longer need to define crossover and or mutation.

**Algorithm 1:**
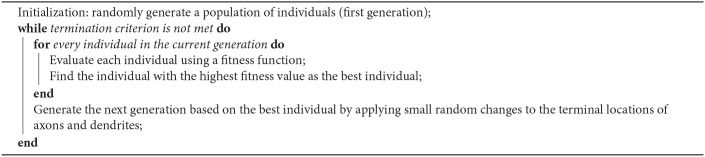
Proposed Genetic Algorithm

In order to obtain the best individual for a classification problem, a fitness function is defined by which the performance of each individual is evaluated. Algorithm 2 represents the evaluation method applied to compute the fitness values of individuals. In order to evaluate each individual, a layer called the readout layer needs to be added to the proposed model. For example, in N-MNIST classification problem, this layer consists of 10 neurons each of which represents a class. The neurons of ionic liquid layer are fully connected to the neurons of the readout layer. *N* and *C* represent the number of neurons in ionic liquid space and the neurons of readout layer, respectively.

**Algorithm 2:**
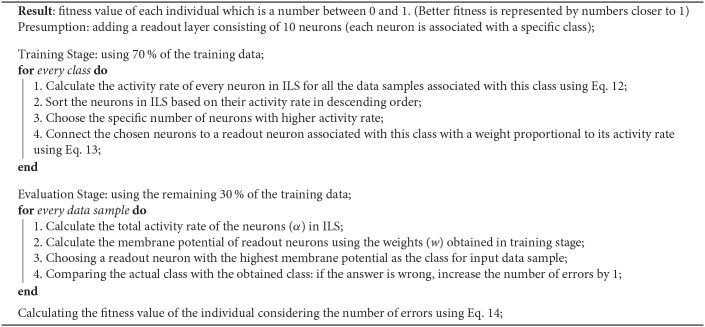
The Algorithm for Evaluating an Individual

In order to set the weight values of this layer, 70% of the training data is used. In this training algorithm, after injecting the data of each class into the ILS, the activity rate of the neurons inside ILS are obtained. The activity rate of the *ith* neuron inside ILS for a given data sample is computed using Equation (11) in which *T* represents the total number of time steps and the activity status of the *ith* neuron in *t* time step is represented by $x_{i}^{t}$. By applying all the data samples of each class, the total activity rate of the *ith* neuron (α_*i*_) is computed using Equation (12) in which $a_{i}^{s}$ represents the activity rate of the *ith* neuron for the *s*^*th*^ data sample of a class. The specific number of neurons which are more active compared to other neurons in ILS are connected to the readout neuron associated with that class with a weight value proportional to their activity. The weight value of less active neurons is set to 0. By assuming *A*^*c*^ as the set of the most active neurons for the *cth* class, the weight between the *cth* readout neuron and the *ith* neuron inside ILS is computed using Equation (13). If the *ith* neuron inside ILS (*N*_*i*_) is a subset of *A*^*c*^, the weight is computed based on $\alpha_{i}^{c}$ while it is set to 0 if *N*_*i*_ is not a subset of *A*^*c*^.

(11) $$\begin{array}{r} a_{i}=\frac{1}{T}\overset{T}{\underset{t=1}{\sum }}x_{i}^{t} \end{array}$$

(12) $$\begin{array}{r} \alpha_{i}=\frac{1}{S}\overset{S}{\underset{s=1}{\sum }}a_{i}^{s} \end{array}$$

(13) wic = {αic−1C−1∑j=1, j≠cCαicNi⊂Ac0Ni⊂Ac

In evaluation stage, using the weight values obtained in training stage, the remaining 30% of the training data is classified. The neuron in the readout layer with the highest membrane potential represents the class of input data. Next, the number of total errors which occurred while classifying this remaining 30% of training data is computed. Using Equation (14), a fitness value is calculated for each individual. In this equation, *D*_*s*_ and *E*_*s*_ indicate the desired and estimated class of the *sth* data sample of the remaining 30% of the training data, respectively. Also, in this equation, *S* represents the number of data samples in the remaining 30% of the training data. Higher fitness values represent better individuals. In summary, this section provided an explanation on how to obtain the best topology of a neural network in ILS for classification problem using optimization algorithm.

(14) $$\begin{array}{r} Fitness=1-\frac{1}{S}\overset{S}{\underset{s=1}{\sum }}\left(D_{s}\neq E_{s}\right) \end{array}$$

## 5. Experimental Results

In this section, we first show the classification results on N-MNIST dataset using the optimized ionic liquid. Then, optimized ionic liquid is compared to the original LSM via separation and classification accuracy considering the same readout learning rule. Also, the performance of the proposed model in comparison with some reservoir models is studied via classification problems derived from the well-known TIMIT dataset (Garofolo et al., 1993). Finally, the scalability, stability and robustness to noisy input of the proposed ionic liquid are discussed.

### 5.1. Classification Results on N-MNIST Dataset by Optimizing the Topology of the Proposed Reservoir

**Figure 6:**
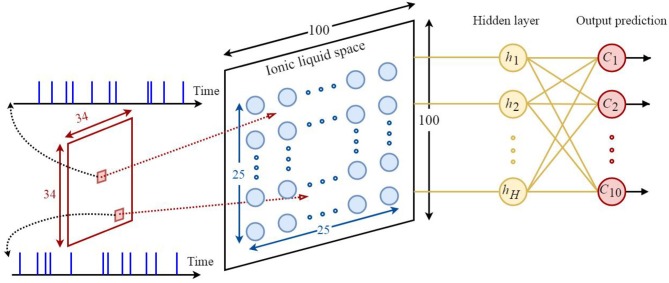
The architecture of the proposed neural network based on the ILS model used to classify the N-MNIST dataset. Each sample consists of 34×34 = 1156 pixels. Each pixel is represented by a 300*ms* event stream injected into an ILS bin. To show the states of the ILS, 25 × 25 = 625 neurons were considered in a 100×100 ILS. In this network the readout layer is a two-layered fully-connected structure consisting of hidden layer neurons and output prediction neurons. There are 10 neurons for predicting the class of each sample from the dataset. Each neuron represents a specific class. In this paper, the number of hidden layer neurons is considered to be 120.

Based on what was discussed in sections 2 and 3, it could be understood that the proposed model is appropriate for classifying neuromorphic and time variant data since diffusion plays a key role in this model. Hence, the N-MNIST dataset (Orchard et al., 2015)—which is the neuromorphic version of the MNIST dataset—has been used for classification. By applying time variant inputs to the ILS it could be said that the state of ILS is also time variant. The state of the ILS depends on whether the neurons of ILS are active or not. One of the biggest challenges of classification problem using the proposed model is being able to provide a constant output for a time-varying ILS. To do so, similar to all other reservoir networks, we need to add a readout layer to the proposed model. Here, a two-layered fully-connected readout layer similar to the one depicted in Figure 6 is used. In order to train this network, scaled conjugate gradient backpropagation method (SCG) has been used (Møller, 1993).

In this section, the effect of the proposed model's topology evolution on its performance using genetic algorithm is shown. In fact, we would like to show that as the topology of the proposed model evolves, better classification results will be obtained. To do so, we optimize the parameters of the proposed model using a proposed genetic algorithm as well as different experiments in order to realize its effect on the classification accuracy.

**Figure 7:**
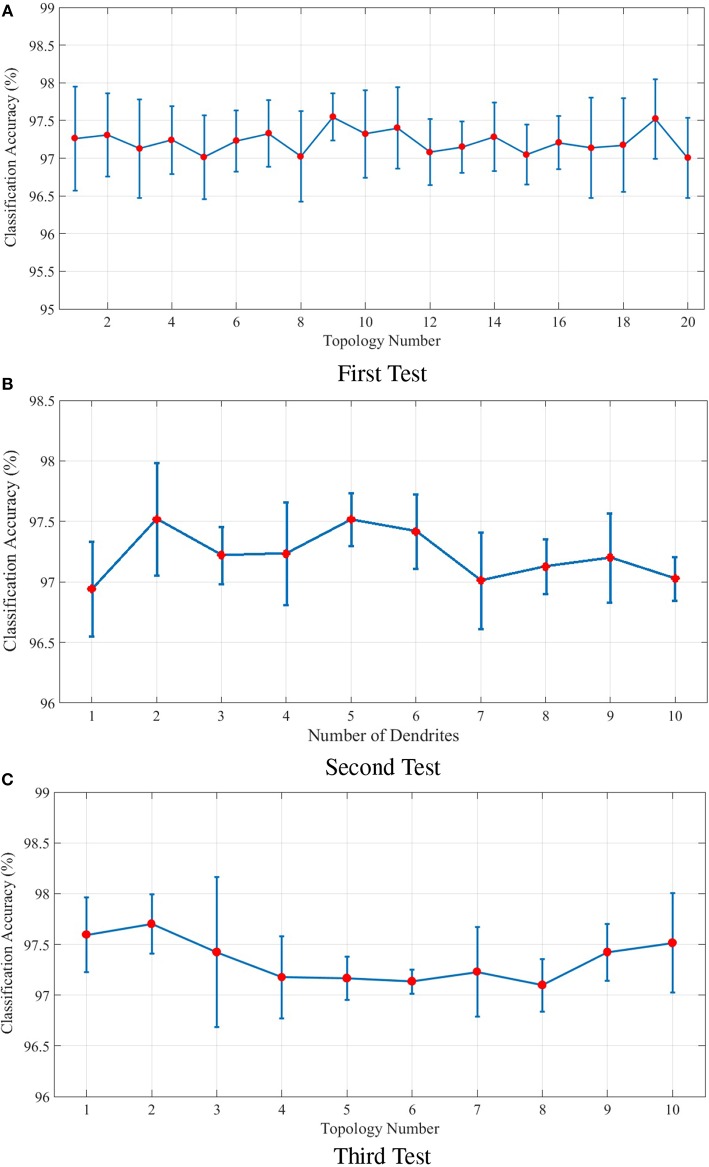
**(A)** Classification results for 20 different topologies generated using the parameters of the first test mentioned in Table 1. In this test, each neuron in the ILS has 4 dendrites. The lengths of dendrites and axons vary for different topologies. The mean and the maximum value of classification accuracy of the topology with the best results are 97.52 and 98.04%, respectively. **(B)** Classification results of the second test considering different number of dendrites for each neuron in the ILS. The best classification results are obtained by considering 2 dendrites per neuron. The mean and the maximum value of classification accuracy by assuming 2 dendrites for each neuron inside ILS are 97.51 and 97.98%, respectively. **(C)** Classification results for ten different topologies generated using the parameters of the third test mentioned in Table 1 and considering two dendrites per neuron. The best mean value for classification accuracy is 97.70% which is obtained by using the second topology and the maximum value of classification accuracy is 98.14% which is associated with the 3*rd* topology. The structure of the proposed ionic liquid space in the 3*rd* topology of this test is considered as the initial individual of the genetic algorithm.

The following includes an explanation on the optimization method used to optimize the topological parameters of the proposed model. In order to optimize the parameters a group of tests are designed. The parameters of each test are shown in Table 1. These parameters include the number of dendrites per neuron as well as the length of dendrites and axons. In the first test, 20 different topologies of the proposed model are built considering the parameters of Table 1. In the first test, 4 dendrites are considered per neuron and the length of dendrites and axons is a random number in a specific range. The results obtained from the classification of the N-MNIST test dataset are depicted in Figure 7A. Since our purpose was to study the topological parameters of the proposed model and not the readout layer, in order to eliminate the effects of the readout layer on classification accuracy, it is trained 10 times for each topology. The classification accuracy obtained in each training is different from the others. Therefore, the diagram has been depicted around the mean values of classification accuracy with confidence intervals.

**Table 1:** Setting parameter values of the proposed model for different tests.

**Test number**	**Number of dendrites**	**Dendrite length**	**Axon length**	**Number of topologies**
First	4	Rand ∈ [0.5, 2]	Rand ∈ [3, 5]	20
Second	1 to 10	1.5	4	10
Third	2	Rand ∈ [0.5, 2.5]	Rand ∈ [3, 6]	10

The second test is designed to evaluate the effect of the number of dendrites on ionic liquid's performance. Therefore, the length of dendrites and axons are set to a constant value and the number of dendrites varies throughout the test. In this test, different topologies are generated by considering a different number of dendrites for each neuron. Similar to first test, the readout layer of the proposed neural network is trained 10 times for each topology. Figure 7B shows the effect of the number of each neuron's dendrites on classification performance. As shown in this figure, better classification accuracy is obtained when the number of dendrites per neuron is set to 2. Hence, in the next test, the number of dendrites per neuron is considered to be 2 as well.

In the third test, each neuron is considered to have 2 dendrites while the length of dendrites and axons is chosen randomly from the ranges given in Table 1. In this test, 10 different topologies of the proposed model are generated. Figure 7C shows the classification results of the third test for different topologies. The best obtained mean value of classification accuracy is 97.70% which is obtained by using the 2*nd* topology while the maximum value of classification accuracy is 98.14% which associates with the 3*rd* topology.

**Figure 8:**
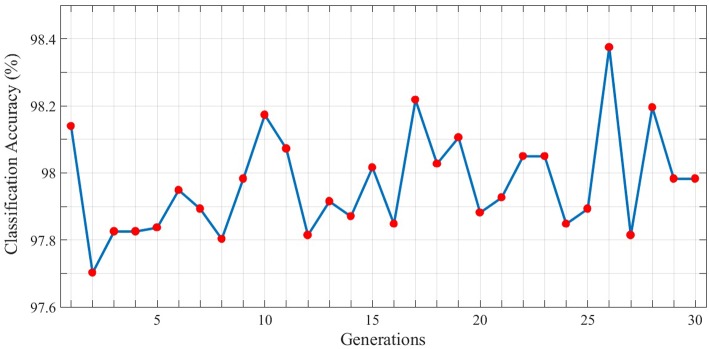
Classification results associated with the best individual in each generation for the N-MNIST test dataset. 30 different generations, each consisting of 10 individuals are generated. The maximum value of classification accuracy is obtained for the 26*th* generation and is equal to 98.38%.

In order to gain better results while using genetic algorithm we use the topology with the maximum classification accuracy. Therefore, the 3*rd* topology of the third test is considered as the initial individual. Now, based on the locations of neurons, dendrites, and axons in this topology, the next generation of individuals will be generated. Each individual is evaluated using the fitness function represented in section 4 and in each generation, the individual with the highest fitness value is used to generate the next population of individuals. The best classification accuracy (best individual) of each generation is depicted in Figure 8. As can be seen, 30 different populations of individuals are generated. As mentioned above, the first population of individuals is generated from the 3*rd* topology of the third test. It also can be seen that the best solution is obtained in the 26*th* generation which holds the maximum value of classification accuracy which is 98.38%.

The results obtained from comparing the proposed model with other state-of-the-art spiking neural networks, while classifying the N-MNIST dataset are shown in Table 2. As can be seen, all the networks used to classify this dataset are in the form of multi-layer perceptron (MLP) and or convolutional neural network (CNN). Different methods are used for training these networks. The highest accuracy of classifying this dataset obtained so far is 98.78% (Wu et al., 2018) in which MLP structure is trained with spatiotemporal backpropagation algorithm (STBP). Network structures are also presented in Table 2. It can be seen that the number of neurons of the proposed model is less than that of other available networks. In other words, using ionic liquid structure leads to obtaining a reasonable classification accuracy with a lesser number of neurons. Without applying the genetic algorithm for optimizing the structure of neurons in the ILS and only by applying the SCG training method in order to train the readout layer of the proposed model, a classification accuracy of 97.69% is obtained. Optimizing the structure of neurons using the genetic algorithm, however, leads to obtaining a classification accuracy of 98.38%.

**Table 2:** Comparing the proposed model with other state-of-the-art spiking neural networks while classifying the N-MNIST dataset.

**Model**	**Network structure**	**Learning-rule**	**CA (%)**
MLP (Wu et al., 2018)	34 × 34 × 2−800−10	STBP	98.78
MLP (Lee et al., 2016)	34 × 34 × 2−800−10	BackProp	98.66
CNN (Stromatias et al., 2017)	18 × 22 × 22−2178−10	SGD	97.77
CNN (Neil and Liu, 2016)	−	−	95.72
MLP (Cohen et al., 2016)	34 × 34 × 2−10000−10	OPIUM (van Schaik and Tapson, 2015)	92.87
The proposed model	25 × 25−120−10	SCG	97.69
The proposed model	25 × 25−120−10	SCG & GA	98.38

### 5.2. Comparison With Other Reservoir Models

In order to show the performance of the proposed model in the N-MNIST classification problem compared to the original LSM architecture, we used CSIM (Natschläger et al., 2003) to implement a spike-based reservoir. We used the StaticSpikingSynapse model for synapses, and the SpikingInputNeuron and LifNeuron for the input and reservoir neurons, respectively. Table 4 contains more details about the LSM parameters in this experiment. The mean separation of the proposed liquid in comparison with the original LSM is then computed. Our model with and without topological optimization provides 18% and 11% increase in liquid separabilities, respectively compared to the original LSM with static synapses. Table 3 contains the mean separation of the original LSM and the proposed model on N-MNIST dataset. Since the number of neurons affects the separation value, the same number of neurons (25 × 25) for both the original LSM and the proposed model is considered in this experiment. To obtain the accuracy of classification, the readout layer consisting of multiple perceptrons each for a class, is considered and then trained using p-delta-rule. Table 3 also contains the mean classification accuracy of the original LSM and the proposed model on N-MNIST test dataset. It is clearly seen that the proposed liquid outperforms the original LSM. Moreover, using a genetic algorithm to optimize the network topology in ionic liquid not only leads to increasing the accuracy, but also increases the separation capability.

**Table 3:** The N-MNIST classification results of proposed liquid compared to the LSM.

**Model**	**Liquid structure**	**Learning rule of readout**	**Learning rule of liquid**	**Mean separation**	**Mean accuracy (%)**
LSM	25 × 25	p-delta-rule	–	0.55	90.87
Ionic liquid			–	0.62	91.48
Ionic liquid			GA	0.67	92.56

**Table 4:** The specifications of the LSM and the proposed liquid.

**LSM specification parameters**	
Neuron type	LIF neuron^a^ (lifNeuron)
Number of neurons	Different for each problem (*N* = *L* × *L*)
Synaptic type	Static Synapses (StaticSpikingSynapses)
Percentage of excitatory neurons	80
Percentage of inhibitory neuron	20
Internal connectivity (λ)	2.5
**Proposed liquid specification parameters**	
Neuron type	Adaptive-threshold LIF neuron
Number of neurons	Different for each problem (*N* = *L* × *L*)
Size of liquid space	(4*L* × 4*L*)
Intrinsic plasticity rate (η)	About 0.2
Transmitted flow coefficient α	0.1
Leakage coefficient (β)	0.05
Fraction of active neurons at any time ($\frac{k}{N}$)^b^	Small about 0.05

a *The parameters of LIF neuron are set to the values similar to those in Table 1 of Roy and Basu (2016)*.

b *Typically we choose k≪N which ensures population sparseness, i.e., only a small fraction of neurons are active in the liquid at any given time*.

**Figure 9:**
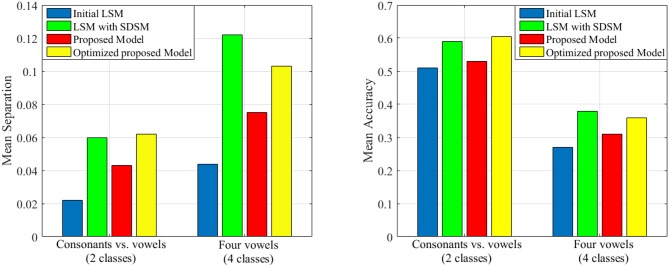
The separation and accuracy of the proposed model in comparison with traditional LSM and SDSM across two problems derived from TIMIT dataset. The mean value of separation and accuracy are obtained for 50 liquids.

As it is mentioned in the introduction, there are a lot of studies that focus on improving the liquid performance. In this section, we tend to compare the performance of our proposed model with that of other works including the random generated liquid (traditional LSM) and the improved liquid using separation driven synaptic modification (SDSM) (Norton and Ventura, 2010) for two classification problems derived from TIMIT dataset (Garofolo et al., 1993). To compare our results, the total number of neurons in the proposed model is considered the same as the number of neurons in Table 2 of Norton and Ventura (2010). Also, we use perceptron for training the readout layer similar to Norton and Ventura (2010). The mean accuracy and mean separation of the proposed model when using TIMIT dataset (Garofolo et al., 1993) are shown in Figure 9. TIMIT dataset consists of 6300 spoken sentences, sampled at 16*kHz*. This dataset consists of 52 phonemes which makes it difficult to correctly identify all of them. Hence, Norton and Ventura (2010) reduces the problem to two simpler problems: The first is identifying phonemes as either “vowels” or “consonants,” and the other is identifying one of four “vowel” phonemes. In this experiment, we use the same method for converting phoneme WAV to spike trains as used in Norton and Ventura (2010). It is clearly seen that the proposed liquid performs better than the initial LSM in cases of either mean separation or mean accuracy for the testing patterns. Also, it is clearly seen that the separation and the accuracy of the proposed model optimized by genetic algorithm are better than that of without topologic optimization. Moreover, it can be seen that the performance of the optimized proposed model and the LSM trained with SDSM learning rule are very close.

### 5.3. Discussion

So far, the efficiency of the proposed model through separation, approximation, and generalization properties were studied. Also, the mean separation and mean accuracy for several classification problems confirmed the better performance of the proposed liquid compared to the random generated LSM. This subsection discusses some of the other capabilities of the proposed model such as scalability, stability and robustness to noisy input. Scalability of a neural network model is an attribute that describes its ability to grow and manage other demands. Hence, in order to show the scalability of the model, the proposed model was used to classify different types of datasets consisting of FSDD, TIMIT, and N-MNIST. To classify each dataset, different numbers of neurons in liquid were considered depending on the difficulty of the problem. It is also clear from the results that the proposed model can function properly with its liquid growth depending on the difficulty of the problem.

In order to explain how the inclusion of intrinsic plasticity helps in stabilizing the learning in ILS, the separation property of the proposed model should be carefully studied. As can be seen in Equation (10), separation is directly proportional to inter-class distance (*c*_*d*_) while it is inversely proportional to intra-class variance (*c*_*v*_). High separation results in better effectiveness of the model. Therefore, high inter-class distance and low intra-class variance are desirable. With low inter-class distance, the liquid behaves in a particular way regardless of the input class. That is because particular ionic trajectories are formed and crystalized in ILS which may result in activating particular neurons. The crystalized ionic trajectories act like strong synapses, leading to the liquid behaving in a particular way without considering the input class. It is such like that the input does not affect the liquid anymore. To rectify, it is necessary to increase the chaotic characteristic of the liquid. Intrinsic plasticity adjusts the neurons' thresholds to ensure homeostasis of their activity. Neurons which receive high levels of input driving them to activation, are more likely to raise their thresholds while neurons receiving lower levels of input lower theirs. This results in the increase of the chaotic level of the proposed model. As a result, the power to distinguish between different classes grows. Also, it makes the intra-class variance increase. Therefore, in order to achieve high inter-class distance and low intra-class variance, a trade-off between diffusion and IP has to be established.

In addition, robustness against noisy or shifted input is one of the advantages of the proposed model due to the effect of diffusion in ionic liquid. What influences the performance of the proposed model is the overall effect of the input data over time. Although the current input data causes the ionic density of the liquid to change which results in changing the internal liquid state, the preceding inputs have affected the ionic density. It means ionic density of ILS depends on both the current and the preceding inputs. This property works like low pass filter which results in less sensitivity to noisy input. For example, assuming the spike train as the input, if the spike occurs sooner than the predefined time, this spike affects ionic density. However, the effect of this spike diminishes. Conversely, if the spike occurs later, its effect on the ionic density increases. Robustness against noisy or shifted input was shown in section 3 while talking about generalizability of the model.

## 6. Conclusion

In this paper, a new model of reservoir networks has been introduced. The synaptic plasticity of this model leads to better learning capability. In this model, neurons are located in an ionic liquid space in which the changes of ionic density result in the neurons being fired. What makes this model different from traditional neural networks is the connection of neurons in ionic liquid space. In fact, the natural diffusion in ionic liquid space results in the creation of ionic trajectories which represent the connection of neurons. In this paper, we first showed the separation, approximation and generalization capabilities of the proposed ionic liquid by performing some experiments consisting of classifying free-spoken-digit-dataset which shows better performance of the proposed model than that of the randomly generated LSM. Then we put our focus on optimizing the topology of the proposed model or the structure of a spiking neural network in ILS. For a specific problem, the topology of the proposed model should be optimized in a way that the performance of the network improves. Since the proposed model is suitable for classifying neuromorphic and or spatiotemporal data, it has been applied for classification of the N-MNIST dataset. In order to optimize the topology of the proposed model for classifying this dataset, first, several tests have been conducted to fix some of the parameters. Then, considering these parameters and by applying the genetic algorithm, the best possible topology has been obtained. Without optimizing the topology of the proposed model and only by applying a training algorithm to the readout layer, the maximum value of classification accuracy (97.69%) for N-MNIST dataset has been obtained. However, optimizing the topology of the proposed model has remarkably increased the classification accuracy on this dataset to 98.38%. Based on the results obtained from topological optimization, it can be concluded that optimizing the structure of neurons in ionic liquid space results in better classification. In addition, the comparison results via classifying the two problems derived from the TIMIT dataset showed that the optimized proposed model outperforms the original LSM and it is comparable with LSM trained with some learning rules.

## Data Availability Statement

All datasets used are publicly available.

## Author Contributions

EI designed the proposed model, carried out the implementation of the proposed model, performed some experiments, analyzed the results, and wrote the manuscript. SS supervised the project and contributed to the final version of the manuscript. MF contributed to the design and implementation of the proposed model. NB helped to write the manuscript. BL-B helped supervise the project and provided helpful suggestions to evaluate the proposed model.

### Conflict of Interest

The authors declare that the research was conducted in the absence of any commercial or financial relationships that could be construed as a potential conflict of interest.

## Footnotes

^1^A class of functions *F* has the approximation property if for any *n* ∈ *N*, any closed set *X* ⊆ **R**^*n*^, any continuous function *h*:*X* → **R**, and any given ρ > 0, there exist some *f* ∈ *F* so that |*h*(*x*) − *f*(*x*)| ≤ ρ, ∀*x* ∈ *X*.
